# Osteopontin Overexpression Induced Tumor Progression and Chemoresistance to Oxaliplatin through Induction of Stem-Like Properties in Human Colorectal Cancer

**DOI:** 10.1155/2015/247892

**Published:** 2015-05-27

**Authors:** Lui Ng, Timothy Wan, Ariel Chow, Deepak Iyer, Johnny Man, Guanghua Chen, Thomas Chung-Cheung Yau, Oswens Lo, Chi-Chung Foo, Jensen Tung-Chung Poon, Ronnie Tung-Ping Poon, Roberta Pang, Wai-Lun Law

**Affiliations:** ^1^Department of Surgery, Li Ka Shing Faculty of Medicine, The University of Hong Kong, Hong Kong; ^2^Centre for Cancer Research, Li Ka Shing Faculty of Medicine, The University of Hong Kong, Hong Kong

## Abstract

Colorectal cancer (CRC) is one of the most common and fatal malignancies worldwide. The poor prognosis of colorectal cancer patients is due to development of chemoresistance and cancer metastasis. Recently osteopontin (OPN) has been associated with stem-like properties in colorectal cancer. This study further examined the clinicopathological significance of OPN in CRC and its effect on chemoresistance and transcription of stem cell markers. We examined the transcription level of OPN in 84 CRC patients and correlated the expression with their clinicopathological parameters. The associations of OPN overexpression with transcription of stem cell markers and response to chemotherapy in DLD1-OPN overexpressing clones and CRC patients were also investigated. Our results showed that OPN was significantly overexpressed in CRC, and its overexpression correlated with tumor stage and poor prognosis. Overexpression of CRC induced OCT4 and SOX2 expression* in vitro* and correlated with SOX2 overexpression in CRC patients. In addition, DLD1-OPN overexpressing cells showed enhanced ability to survive upon oxaliplatin treatment, and OPN expression was higher in CRC patients who were resistant to oxaliplatin-involved chemotherapy treatment. Thus, CRC cells overexpressing OPN demonstrated stem-like properties and OPN inhibition is a potential therapeutic approach to combat CRC progression and chemoresistance.

## 1. Introduction

Colorectal cancer (CRC) is the third most common malignancy around the world [1]. Annually, over 1.2 million people develop CRC globally, with more than 600,000 patients dying from the disease in 2008 [2]. Both the incidence and the death rates from CRC are increasing rapidly in Asian countries [3]. Incidence and mortality rates for CRC have declined as a result of improved tests that allow early detection of the cancer, when it can be more easily treated by surgery and chemotherapy along with radiotherapy [4]. Despite those advances in clinical treatment, the overall prognosis of CRC patients is still unsatisfactory due to development of chemoresistance and cancer metastasis. Therefore, it is important to understand how CRC cells acquired the ability to survive upon chemotherapy and metastasize to distant regions, in order to develop new therapeutic target and approach to improve the prognosis and survival rates of CRC patients.

Osteopontin (OPN), a member of the Small Integrin-Binding Ligand N-linked Glycoprotein (SIBLING) family, is expressed in normal mineralized tissues, epithelial cells of some metabolically active ducts, and several neoplastic tissues [5]. OPN is involved in most aspects of tumor biology. Through its diverse reported functions related to proliferation, survival, angiogenesis, escape from host defense, tumor development, invasion, and metastasis, OPN covers multiple hallmarks of cancer [6]. Indeed, OPN expression significantly correlates with tumor stage in various cancer types.

In colorectal cancer, OPN downregulation suppressed* in vitro* proliferation and* in vivo* tumorigenicity and also suppressed* in vitro* invasion and migration capacity [7]. We also showed in our previous study that stable overexpression of OPN in DLD1 cells significantly induced the protein expression and secretory level of OPN, and the migration ability of DLD1 cells [8]. In addition, OPN overexpression is associated with activation of the epithelial to mesenchymal pathway through induction of Twist and Snail and downregulation of E-cadherin.

Recently, OPN has been associated with cancer stem cell nature in colorectal cancer. OPN secreted from tumor associated cells increased CD44v6 expression in colorectal cancer stem cells by activating the Wnt/*β*-catenin pathway, which promotes migration and metastasis [9]. This study will further investigate the* in vitro* effect of OPN overexpression on growth response to chemotherapy. In addition, a recent study demonstrated that OPN silencing suppressed transcriptions of key stemness transcription factors SOX2, Oct3/4, and Nanog* in vitro* and glioblastoma stem-like cell character and tumorigenicity* in vivo* [10]. This study will also study the effect of OPN overexpression on stemness of CRC cells, by investigating its correlation with transcription of stem cell markers.

## 2. Materials and Methods

### 2.1. Patients and Specimens

The human sample collection protocol has been approved by the Institutional Review Board (IRB) of the University of Hong Kong, and all clinical investigation has been conducted according to the principles expressed in the Declaration of Helsinki. Informed written consent has been obtained from the participants. Tissue samples were obtained from 84 patients, immediately frozen in liquid nitrogen and kept at −80°C until analysis. Clinicopathological data were obtained from the patient database of our hospital.

### 2.2. Cell-Lines, Tissue Culture, Transfections, and Reagents

Construction of stable OPN overexpressing or vector control DLD1 cells was described previously [8]. DLD1-OPN stable clones and vector control were maintained in DMEM with 10% fetal bovine serum and antibiotics (Invitrogen, Carlsbad, CA), 5% CO_2_ at 37°C.

### 2.3. RNA Extraction

Total RNA was extracted using Trizol reagent and Purelink RNA Mini Kit (Life Technologies, Carlsbad, CA) according to the manufacturer's instructions. The RNA yield and quality were analyzed by NanoDrop 2000 (Thermo Scientific).

### 2.4. cDNA Synthesis and Quantitative Real-Time Polymerase Chain Reaction

500 ng total RNA was reversely transcribed with PrimeScript 1st strand cDNA Synthesis Kit (Takara, Tokyo, Japan) in accordance with the instructions of the manufacturer. Real-time PCR was performed in a final volume of 15 *μ*L containing 1.5 *μ*L RT transcript, 0.2 *μ*M of each primer, 1x ROX reference dye, and 7.5 *μ*L of FastStart Universal SYBR Green Master (ROX) (Roche Diagnostics, Switzerland, Basel). A no RT transcript control was included for each gene to ensure the signal was truly driven by target gene amplification. The primer sequences were as follows: OPN-Forward Primer: 5′-TGGGGGTCACTGCAATTAG-3, OPN-Reverse Primer: 5′-TGGGGCTAGGAGATTCTG-3′; GAPDH-Forward Primer: 5′-GTCTCCTCTGACTTCAACAGCG-3′, GAPDH-Reverse Primer: 5′-ACCACCCTGTTGCTGTAGCCAA-3′; OCT4-Forward Primer: 5′-GTGGAGGAAGCTGACAACAA-3, OCT4-Reverse Primer: 5′-GCCGGTTACAGAACCACACT-3′; SOX2-Forward Primer: 5′-GACAGTTACGCGCACATGAA-3, SOX2-Reverse Primer: 5′-TAGGTCTGCGAGCTGGTCAT-3′; Nanog-Forward Primer: 5′-GTGATTTGTGGGCCTGAAGA-3, Nanog-Reverse Primer: 5′-ACACAGCTGGGTGGAAGAGA-3′. Real-time PCR was carried out using the ABI 7900HT Fast Real-Time PCR System (Applied Biosystems, Foster, CA) at 95°C for 10 min, followed by 40 cycles at 95°C for 15 sec and at 56°C for 1 min. Each assay was done in triplicate, the average was calculated, and the expression level of target mRNA was normalized by the expression of GAPDH (delta Ct); that is, the higher the delta Ct, the lower the target gene expression.

### 2.5. Cell Viability Assay

Same number of cells were seeded on 96-well plate for 24 hours and then subjected to treatment of chemotherapeutic drugs 5 *μ*M oxaliplatin or 50 *μ*M 5-Fluorouracil (5FU). The cell viability at 72 h after drug treatment was determined using the 3-[4,5-dimethylthiazol-2-yl]-2,5-diphenyl-tetrazolium bromide (MTT) (Invitrogen).

### 2.6. Immunohistochemical Staining

Immunohistochemical staining was performed as described previously [11]. Sections were incubated with the primary antibodies anti-OPN (OriGene Technologies, Rockville, MD) and anti-SOX2 (Cell Signaling Technology, Danvers, MA) at 1 : 100 dilutions overnight at 4°C in a moist chamber. A scoring system related to the extent and intensity of immunostaining of enterocytes was used. The intensity of positive staining was scored as 0, negative; 1, weak; 2, moderate; 3, strong by two independent observers. The extent of positive staining was scored as 1 (<10%), 2 (10–50%), and 3 (>50%). The final score was determined by multiplying the intensity score and extent score, yielding a range from 0 to 12.

### 2.7. Statistical Analysis

The association of OPN level with stem cell markers was tested with Pearson correlation. Student's *t*-test was applied to compare difference between two groups. Survival rates were analyzed with log-rank test. All of these statistical analyses were performed with SigmaPlot 10.0 (Systat Software Inc., San Jose, CA, USA). Statistical significance was set at *p* ≤ 0.05.

## 3. Results

### 3.1. OPN Was Overexpressed in CRC

We first compared the expression of OPN in CRC and the paired nontumor mucosa of 84 CRC patients. The OPN expression was determined by qRT-PCR and expressed as fold change to the adjacent nontumor tissue using the 2^−ΔΔCt^ method. Our results showed that the mean expression of OPN in CRC was significantly higher than that in the nontumor tissue (7.4-fold, *p* < 0.001).

### 3.2. OPN Overexpression Correlated with Advanced Tumor Stage

**Figure 1:**
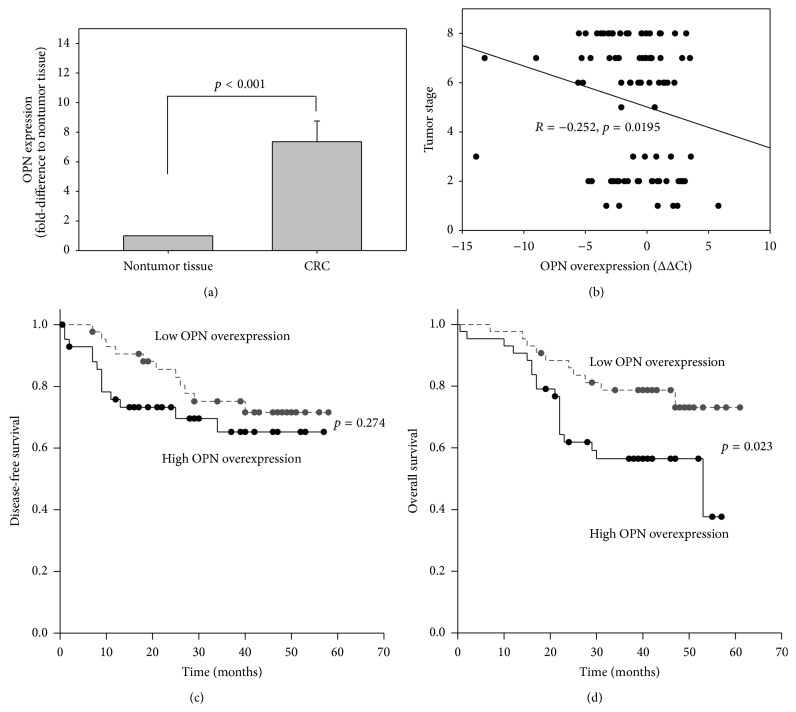
(a) Relative transcript expression of OPN (expressed as fold change to the adjacent nontumor tissue using the 2^−ΔΔCt^ method) of 84 patients; correlation of OPN overexpression (expressed as ΔΔCt (ΔCt of CRC −ΔCt of nontumor)) with (b) tumor stage, (c) disease-free survival, and (d) overall survival of CRC patients.

We next examined the clinicopathological significance of OPN overexpression (expressed as ΔΔCt (ΔCt of CRC, ΔCt of nontumor tissue), that is, lower ΔΔCt value represented higher OPN overexpression in CRC) in 84 CRC patients (Table 1). OPN overexpression was not associated with age, gender, and tumor size, but there was a trend of higher OPN overexpression in patients with lymph node metastasis (*p* = 0.057) and distant metastasis (*p* = 0.088). In addition, when we divided the patients into lower stage (no lymph node and distant metastasis) and higher stage (presence of lymph node and/or distant metastasis), patients of higher CRC stage showed significantly higher OPN overexpression (*p* = 0.014). OPN overexpression was also significantly correlated with tumor stage (*R* = −0.252, *p* = 0.0195; Figure 1(b)). Moreover, our results showed that OPN overexpression correlated with the grade of CRC. Poorly differentiated CRC that tend to be more aggressive showed higher OPN overexpression when compared with well or moderately differentiated CRC (*p* = 0.02). These results suggested that OPN overexpression correlated with disease progression of CRC.

### 3.3. OPN Overexpression Correlated with Poor Prognosis

The associations of OPN overexpression with disease-free survival (DFS) and overall survival (OS) were investigated. We divided the 84 CRC patients into 2 groups according to the OPN overexpression status of their CRC. For patients whose OPN overexpression was below the median level of the 84 patients (ΔΔCt > −0.617), they were regarded as low OPN overexpression. On the other hand, for those whose OPN overexpression was above the median level (ΔΔCt ≤ −0.617), they were regarded as high OPN overexpression. We first compared the DFS rates between the two groups of patients. Though high OPN overexpression patients appeared to have a worse DFS than that of weak overexpression group, the difference was not statistically significant (*p* = 0.274; Figure 1(c)). On the other hand, patients with low OPN overexpression have a significantly better OS rate than patients with high OPN overexpression (*p* = 0.023; Figure 1(d)). These results suggested that OPN overexpression correlated with poor prognosis in CRC patients.

### 3.4. Correlation of OPN Overexpression with Stem Cell Marker Levels

**Figure 2:**
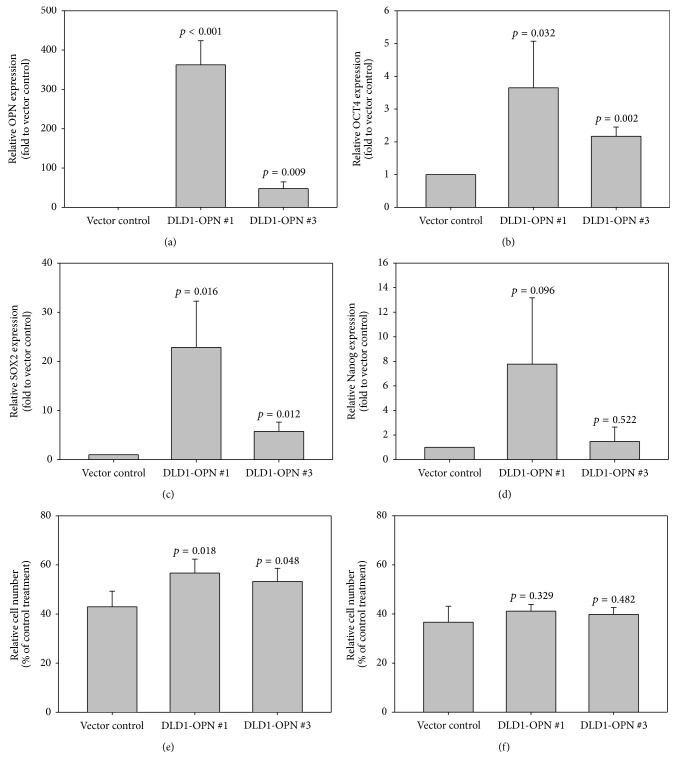
Relative overexpression (expressed as ΔΔCt (ΔCt of CRC −ΔCt of nontumor)) of (a) OPN, (b) OCT4, (c) SOX2, and (d) Nanog in DLD1-OPN stable clones #1 and #3 and the vector control; the relative number of viable cells (expressed as percentage of viable cells when compared with vehicle treated cells) after 72-hour treatment of (e) 5 *μ*M oxaliplatin and (f) 50 *μ*M 5FU.

To investigate the effect of OPN overexpression on transcription of stem cell markers, we determined the transcription level of OCT4, SOX2, and Nanog [12] in two DLD1-OPN overexpressing cells DLD1-OPN #1 and #3 and the vector control DLD1-Vc [8] by qRT-PCR. As shown in Figure 2(a), OPN transcript expression of DLD1-OPN #1 and 3 was significantly higher than that of vector control. The expressions of OCT4 and SOX2 were also significantly higher in DLD1-OPN stable clones when compared with the vector control (Figures 2(b) and 2(c)), suggesting that overexpression of OPN induces the transcription of OCT4 and SOX2. In addition, the induction effect of OPN overexpression on SOX2 was higher than that on OCT4. On the other hand, though Nanog expression appeared higher in DLD1-OPN stable clones than in vector control, the difference was not statistically significant (Figure 2(d)).

**Figure 3:**
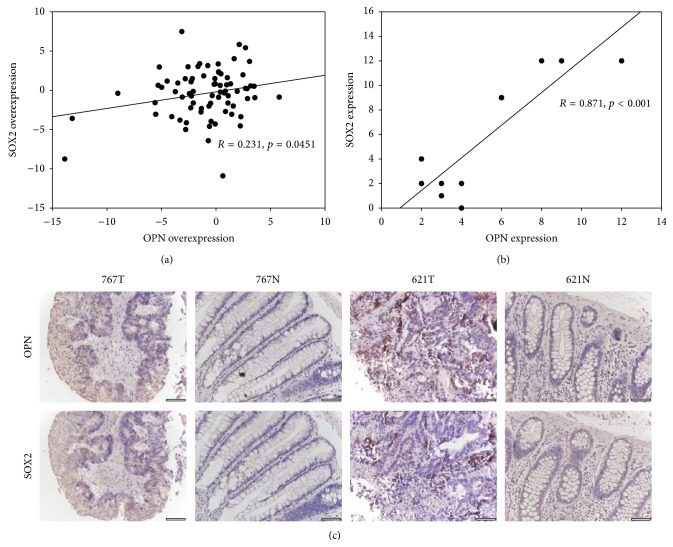
(a) Correlation of OPN mRNA overexpression with SOX2 mRNA overexpression in CRC patients. (b) Correlation of OPN protein expression with SOX2 protein expression in CRC tissues of 11 patients. (c) Representative images showing correlation of OPN and SOX2 protein expression in CRC tissues (621T: OPN high/SOX2 high; 767T: OPN low/SOX2 low). Negative/weak staining of OPN and SOX2 were detected in most adjacent normal tissues (N).

We next examined the correlation of OPN with OCT4, SOX2, and Nanog in the CRC patients. Overexpression of OPN in CRC positively correlated with SOX2 overexpression (*R* = 0.231, *p* = 0.0451; Figure 3(a)), suggesting that tumor cells with high OPN expression possess cancer stem cell-like properties. On the other hand, OPN overexpression in CRC did not correlate with Nanog overexpression (*R* = 0.009, *p* = 0.940), which is in accordance with the result obtained in our cell-line experiment. Furthermore, overexpression of OPN in CRC did not correlate with OCT4 overexpression (*R* = −0.171, *p* = 0.173), which is not in accordance with our cell-line data.

We further investigated the correlation of OPN and SOX2 protein expression in 11 CRC patients using immunohistochemistry. There was a positive correlation between OPN and SOX2 expression in the CRC specimen tested (*R* = 0.871, *p* < 0.001; Figures 3(b) and 3(c)).

### 3.5. OPN Overexpression Induces Chemoresistance to Oxaliplatin Treatment

OPN overexpression was associated with chemoresistance in small-cell lung cancer, breast cancer, and glioma [13–15]; yet its role in CRC chemoresistance has not been demonstrated. To examine the effect of OPN overexpression on chemoresistance of CRC cells, we treated the DLD1-OPN stable cells and vector control with oxaliplatin and 5-FU which are common chemodrugs to treat CRC patients and determined the relative number of viable cells after 72 hours by MTT assay. The effect on CRC cell viability was expressed as percentage of viable cells when compared with vehicle treated cells. As shown in Figure 2(e), our results showed that following 5 *μ*M oxaliplatin treatment, the percentage of viable vector control cells (43.0%) was significantly lower than that of DLD1-OPN #1 (56.7%, *p* = 0.018) and DLD1-OPN #3 (53.3%, *p* = 0.048). On the other hand, OPN overexpression did not enhance the chemoresistance of DLD1 cells to 50 *μ*M 5FU treatment (Figure 2(f)).

We also compared the OPN overexpression in CRC patients who received chemotherapy. 55 patients with detailed clinical information after administration of chemotherapy treatment were included in this comparison. Among these patients, 29 of them were sensitive to the treatment (tumor responsive to chemotherapy and no recurrence within at least 1 year) while 26 of them were resistant (tumor not responsive to chemotherapy or recurrence within 1 year). The chemoresistance patients showed higher OPN overexpression when compared with the chemosensitive patients (−2.427 versus −0.385, *p* = 0.013).

## 4. Discussion

Previously, involvement of OPN in most aspects of tumor biology has been reported. Through its diverse reported functions related to proliferation, survival, angiogenesis, escape from host defense, tumor development, invasion, and metastasis, OPN covers multiple hallmarks of cancer [6]. Indeed, OPN expression significantly correlates with tumor stage in various cancer types.

In colorectal cancer, OPN downregulation suppressed* in vitro* proliferation and* in vivo* tumorigenicity and also suppressed* in vitro* invasion and migration capacity [7]. We also showed in our previous study that stable overexpression of OPN in DLD1 cells significantly induced the protein expression and secretory level of OPN, and the migration ability of DLD1 cells [8]. In this study, we further examined the clinicopathological significance of OPN expression in CRC and its association with stem-like property and chemoresistance.

We showed that OPN overexpression in CRC correlated with higher grade, tumor stage, and survival of CRC patients, indicating that OPN overexpression was associated with poor prognosis. Stem-like nature of CRC cells has been suggested to associate with tumor progression, metastasis, and poor survival [16, 17], which is in accordance with the clinicopathological characteristics observed in our patients with high OPN overexpression. In addition, OPN has been associated with cancer stem cell in colorectal cancer. OPN secreted from tumor associated cells increased CD44v6 expression in colorectal cancer stem cells by activating the Wnt/*β*-catenin pathway, which promotes migration and metastasis [9]. This study further investigated the effects of OPN overexpression on transcriptions of key stemness transcription factors SOX2, OCT4, and Nanog, in which transcription repression by OPN silencing has been demonstrated in stem-like glioblastoma cells* in vivo* [10]. The expression of OCT4 and SOX2 was significantly higher in DLD1-OPN stable clones #1 and #3, when compared with the vector control. In addition, their expression in DLD1-OPN #1 which expressed higher level of OPN was higher than that in DLD1-OPN #3, further indicating that OPN overexpression induced the expression of OCT4 and SOX2, which are associated with the stem-like properties of CRC cells [18]. We next examined the correlation of overexpression of OPN with that of OCT4 and SOX2 in CRC patients. In accordance with our* in vitro* data, OPN overexpression in CRC significantly correlated with SOX2 overexpression. Interestingly, no correlation was observed between OPN and OCT4. As our* in vitro* data showed that DLD1-OPN #1 and #3 demonstrated 22.8-fold and 5.7-fold induction of SOX2, respectively, whereas only 3.6-fold and 2.2-fold induction of OCT4 was detected in DLD1-OPN #1 and #3, respectively, we suggest that the correlation of OPN with SOX2 is stronger than that with OCT4 and thus only such correlation was observed in our patient cohort. Nonetheless, as SOX2 was known to be a stem cell factor in CRC which enhanced CRC cell proliferation, migration, and invasion [19, 20], our results indicated that OPN overexpression induced stem-like properties of CRC cells through overexpression of SOX2.

This study also demonstrated that DLD1-OPN stable clones showed improved survival rate upon oxaliplatin treatment. In addition, DLD1-OPN #1 which showed higher OPN expression than DLD1-OPN #3 was more resistant to the effect of oxaliplatin, suggesting that OPN overexpression indeed regulated such chemoresistance. Previously, OPN downregulation was reported to enhance* in vitro* radiosensitivity of CRC cells [7]. This study further strengthened the unfavourable effects of OPN overexpression on treatment of CRC patients. To our knowledge, this is the first report showing OPN overexpression induced oxaliplatin-chemoresistance. We believed that such drug-resistant effect was associated with the stem cell-like properties in OPN overexpressing cells, as oxaliplatin-resistant CRC cells showed higher levels of stem cell markers SOX2 and OCT4 [21, 22]. We also investigated the effect of OPN overexpression on response of 55 CRC patients to chemotherapy involving oxaliplatin. The mean OPN overexpression of the 26 resistant patients was higher than that of the 29 sensitive patients, suggesting that OPN expression in CRC patients could be a biomarker for predicting the response to chemotherapy involving oxaliplatin. Also, the effect of OPN inhibition on oxaliplatin-based chemotherapy of CRC patients warrants further investigation.

Recently, CD44v6 has been reported as a colorectal CSC marker required for the metastatic potential of CSCs, and OPN, HGF, and SDF-1 increased CD44v6 expression in CSCs [9]. OPN has been suggested to activate the pathway associated with HGF and SDF-1. Ligation of OPN to integrins leads to activation of HGF receptor (Met) and is known to increase the sensitivity of mammary epithelial cell lines to the cell migration promotion effect of HGF/Met [23]. OPN potentiated tumor growth via interaction with mesenchymal stromal cell to upregulate expression of CCL5 and cancer-associated fibroblast markers including SDF-1 [24]. These results showed that OPN overexpression in CRC possibly enhanced the stemness properties of cancer cells through induction of CD44v6 expression, as well as through activation or upregulation of HGF and SDF-1, which are also upregulators of CD44v6.

This study, in combination with the results from other OPN studies (reviewed in [25]), suggested that OPN is a therapeutic target for cancer. Silencing of OPN using RNAi technology, blocking OPN activity using specific antibodies, and small-molecule inhibitors might repress tumor progression and metastasis and enhance the response to chemotherapy in CRC, and possibly in other types of cancer.

## 5. Conclusions

This study demonstrated that OPN overexpression correlated with tumor progression and poor prognosis in CRC patients, possibly by inducing stem-like property of CRC cells as reflected by overexpression of SOX2, which is associated with ability to metastasize and survive upon oxaliplatin-involved chemotherapy treatment.

## Figures and Tables

**Table 1:** Clinicopathological correlation of OPN overexpression in CRC (*N* = 84).

Clinicopathological features	Category	Number of cases	OPN overexpression (ΔΔCt) (mean ± SEM)	*p* value
Age	<65 ≥65	34 50	−0.613 ± 0.475 −0.767 ± 0.371	0.798

Gender	Male Female	59 25	−0.542 ± 0.335 −1.089 ± 0.581	0.394

Tumor size	<5 cm ≥5 cm	49 35	−0.824 ± 0.412 −0.537 ± 0.400	0.630

Histological grade	Well/moderate Poor/undifferentiated	75 9	0.599 ± 0.309 −3.024 ± 1.472	0.020

Lymph node metastasis	Absent Present	37 47	−0.081 ± 0.422 −1.196 ± 0.390	0.057

Tumor AJCC stage	I to II III to IV	31 53	0.219 ± 0.474 −1.245 ± 0.352	0.014

Distant metastasis	Absent Present	64 20	−0.427 ± 0.338 −1.594 ± 0.537	0.088

## References

[1] Shike M., Winawer S. J., Greenwald P. H., Bloch A., Hill M. J., Swaroop S. V. (1990). Primary prevention of colorectal cancer. The WHO Collaborating Centre for the Prevention of Colorectal Cancer. *Bulletin of the World Health Organization*.

[2] Armaghany T., Wilson J. D., Chu Q., Mills G. (2012). Genetic alterations in colorectal cancer. *Gastrointestinal Cancer Research*.

[3] Sung J. J. Y., Lau J. Y. W., Young G. P. (2008). Asia Pacific consensus recommendations for colorectal cancer screening. *Gut*.

[4] Lin J. S., Webber E. M., Beil T. L., Goddard K. A., Whitlock E. P. (2012). Fecal DNA testing in screening for colorectal cancer in average-risk adults.

[5] Bellahcène A., Castronovo V., Ogbureke K. U. E., Fisher L. W., Fedarko N. S. (2008). Small integrin-binding ligand N-linked glycoproteins (SIBLINGs): multifunctional proteins in cancer. *Nature Reviews Cancer*.

[6] Kruger T. E., Miller A. H., Godwin A. K., Wang J. (2014). Bone sialoprotein and osteopontin in bone metastasis of osteotropic cancers. *Critical Reviews in Oncology/Hematology*.

[7] Likui W., Hong W., Shuwen Z., Yuangang Y., Yan W. (2011). The potential of osteopontin as a therapeutic target for human colorectal cancer. *Journal of Gastrointestinal Surgery*.

[8] Ng L., Wan T. M., Lam C. S. (2015). Post-operative plasma osteopontin predicts distant metastasis in human colorectal cancer. *PLOS ONE*.

[9] Todaro M., Gaggianesi M., Catalano V. (2014). CD44v6 is a marker of constitutive and reprogrammed cancer stem cells driving colon cancer metastasis. *Cell Stem Cell*.

[10] Lamour V., Henry A., Kroonen J. (2015). Targeting osteopontin suppresses glioblastoma stem-like cell character and tumorigenicity in vivo. *International Journal of Cancer*.

[11] Ng L., Tung-Ping Poon R., Yau S. (2013). Suppression of actopaxin impairs hepatocellular carcinoma metastasis through modulation of cell migration and invasion. *Hepatology*.

[12] Luo W., Li S., Peng B., Ye Y., Deng X., Yao K. (2013). Embryonic stem cells markers SOX2, OCT4 and Nanog expression and their correlations with epithelial-mesenchymal transition in nasopharyngeal carcinoma. *PLoS ONE*.

[13] Gu T., Ohashi R., Cui R. (2009). Osteopontin is involved in the development of acquired chemo-resistance of cisplatin in small cell lung cancer. *Lung Cancer*.

[14] Graessmann M., Berg B., Fuchs B., Klein A., Graessmann A. (2007). Chemotherapy resistance of mouse WAP-SVT/t breast cancer cells is mediated by osteopontin, inhibiting apoptosis downstream of caspase-3. *Oncogene*.

[15] Qian C., Li P., Yan W. (2015). Downregulation of osteopontin enhances the sensitivity of glioma U251 cells to temozolomide and cisplatin by targeting the NF-kappaB/Bcl2 pathway. *Molecular Medicine Reports*.

[16] Kashihara H., Shimada M., Kurita N. (2014). CD133 expression is correlated with poor prognosis in colorectal cancer. *Hepatogastroenterology*.

[17] Hsu H., Liu Y., Tseng K., Tan B. C., Chen S., Chen H. (2014). LGR5 regulates survival through mitochondria-mediated apoptosis and by targeting the Wnt/*β*-catenin signaling pathway in colorectal cancer cells. *Cellular Signalling*.

[18] Dotse E., Bian Y. (2014). Isolation of colorectal cancer stem-like cells. *Cytotechnology*.

[19] Lu Y.-X., Yuan L., Xue X.-L. (2014). Regulation of colorectal carcinoma Stemness, Growth, and metastasis by an *miR-200c*-Sox2-negative feedback loop mechanism. *Clinical Cancer Research*.

[20] Han X., Fang X., Lou X. (2012). Silencing SOX2 induced mesenchymal-epithelial transition and its expression predicts liver and lymph node metastasis of CRC patients. *PLoS ONE*.

[21] Yang D., Wang H., Zhang J. (2013). In vitro characterization of stem cell-like properties of drug-resistant colon cancer subline. *Oncology Research*.

[22] Wen K., Fu Z., Wu X., Feng J., Chen W., Qian J. (2013). Oct-4 is required for an antiapoptotic behavior of chemoresistant colorectal cancer cells enriched for cancer stem cells: effects associated with STAT3/Survivin. *Cancer Letters*.

[23] Tuck A. B., Chambers A. F., Allan A. L. (2007). Osteopontin overexpression in breast cancer: knowledge gained and possible implications for clinical management. *Journal of Cellular Biochemistry*.

[24] Mi Z., Bhattacharya S. D., Kim V. M., Guo H., Talbotq L. J., Kuo P. C. (2011). Osteopontin promotes CCL5-mesenchymal stromal cell-mediated breast cancer metastasis. *Carcinogenesis*.

[25] Bandopadhyay M., Bulbule A., Butti R. (2014). Osteopontin as a therapeutic target for cancer. *Expert Opinion on Therapeutic Targets*.

